# Distinctive Responsiveness to Stromal Signaling Accompanies Histologic Grade Programming of Cancer Cells

**DOI:** 10.1371/journal.pone.0020016

**Published:** 2011-05-19

**Authors:** Maria Gloria Luciani, Junhee Seok, Aejaz Sayeed, Stacey Champion, William H. Goodson, Stefanie S. Jeffrey, Wenzhong Xiao, Michael Mindrinos, Ronald W. Davis, Shanaz H. Dairkee

**Affiliations:** 1 California Pacific Medical Center Research Institute, San Francisco, California, United States of America; 2 Stanford Genome Technology Center, Stanford University School of Medicine, Stanford, California, United States of America; 3 Department of Surgery, Stanford University School of Medicine, Stanford, California, United States of America; Instituto Nacional de Câncer, Brazil

## Abstract

Whether stromal components facilitate growth, invasion, and dissemination of cancer cells or suppress neoplastic lesions from further malignant progression is a continuing conundrum in tumor biology. Conceptualizing a dynamic picture of tumorigenesis is complicated by inter-individual heterogeneity. In the post genomic era, unraveling such complexity remains a challenge for the cancer biologist. Towards establishing a functional association between cellular crosstalk and differential cancer aggressiveness, we identified a signature of malignant breast epithelial response to stromal signaling. Proximity to fibroblasts resulted in gene transcript alterations of >2-fold for 107 probes, collectively designated as **F**ibroblast **T**riggered Gene **Ex**pression in **T**umor (**FTExT**). The hazard ratio predicted by the FTExT classifier for distant relapse in patients with intermediate and high grade breast tumors was significant compared to routine clinical variables (dataset 1, n = 258, HR – 2.11, 95% CI 1.17–3.80, p-value 0.01; dataset 2, n = 171, HR - 3.07, 95% CI 1.21–7.83, p-value 0.01). Biofunctions represented by FTExT included inflammatory signaling, free radical scavenging, cell death, and cell proliferation. Unlike genes of the ‘proliferation cluster’, which are overexpressed in aggressive primary tumors, FTExT genes were uniquely repressed in such cases. As proof of concept for our correlative findings, which link stromal-epithelial crosstalk and tumor behavior, we show a distinctive differential in stromal impact on prognosis-defining functional endpoints of cell cycle progression, and resistance to therapy-induced growth arrest and apoptosis in low vs. high grade cancer cells. Our experimental data thus reveal aspects of ‘paracrine cooperativity’ that are exclusively contingent upon the histopathologically defined grade of interacting tumor epithelium, and demonstrate that epithelial responsiveness to the tumor microenvironment is a deterministic factor underlying clinical outcome. In this light, early attenuation of epithelial-stromal crosstalk could improve the management of cases prone to be clinically challenging.

## Introduction

It is widely believed that the local microenvironment of host tissue is an active participant throughout cancer development and progression [1]. In this milieu, interactive exchanges between stroma and tumor cells might influence survival, growth, and dissemination [2]. Autocrine stromal chemokines, and paracrine TGFβ signals are well known pro-tumorigenic mediators [3]–[8]. In breast tumors, fibroblasts generally represent the most abundant cell type in the stroma, often referred to as the ‘reactive stroma’, highly enriched in, type I and VI collagen, laminin, entactin, heparan-sulfate proteoglycans and fibrin [9]. Tumor-derived fibroblasts from different organs display considerable variability in transcriptional profiles [10], and significant variations occur in stromal gene expression within tumors of the same organ system, often in association with clinicopathologic parameters. For example, overexpression of CD10, and PDGFß receptor in breast cancer stroma is characteristic of high histologic grade [11], [12]. Fibroblast-associated signatures and their clinical correlation with outcome in cancer patients [13], [14], suggest that an altered tumor stroma is a result of ‘cooperative crosstalk’ with carcinoma cells.

A comprehensive effort to reveal the underpinnings of stromal-epithelial crosstalk within solid tumors could assist in the identification of a broader spectrum of targetable molecular determinants of patient outcome. It is conceivable that aggressive phenotypes of tumor cells often represent a localized response to microenvironmental signals [15]. However, despite transient *in vitro* induction, stromal-epithelial crosstalk has long-lasting functional effects on tumorigenicity and metastatic potential of cells inoculated into experimental animals [16]. In the search for specific phenotypic effects, *in vitro* interactions between fibroblasts and breast epithelial cells previously attempted were limited to spontaneously immortalized breast cancer cell lines uniformly derived from aggressive high grade tumors and metastatic effusions [17]–[19], or from nonmalignant human breast tissue [20]. Interpretation of such data is encumbered by the lack of a wide, disease-based spectrum of surrogate models of human cancer. Thus unequivocal conclusions regarding an association between clinical heterogeneity and the functional biology underlying stromal-epithelial signaling remain elusive. A critical prerequisite for analyzing the interaction between various cellular subsets within tumor tissue is the availability of live interactive components fractionated from fresh surgical discard tissue in quantities sufficient for robust data collection and hypothesis generation. Likewise, to attain translational insights through experimental simulation, it is critically important to incorporate a cellular repertoire, which reflects the breadth of clinical variability contributed by epithelial and stromal elements within tumor tissue. By facilitating direct crosstalk between novel primary tumor cell lines of wide ranging clinicopathology [21] and multiple independent samples of nonmalignant or tumor tissue-derived fibroblasts, our study has aimed towards an improved understanding of cancer biology and its application in the following ways. First, a set of genes is identified, whose expression is consistently altered through paracrine interactions. Next, the role of such a stromally induced tumor cell response in promoting disease severity is illustrated through its correlation with clinical outcome. Finally, a direct functional basis for varying clinical outcome associated with tumor responsiveness to stromal signaling is demonstrated by shifts in cell cycle kinetics, apoptotic evasion, ROS reduction, and neutralization of the growth inhibitory effects of hormonal cancer therapy. Our demonstration of grade-associated variability in the dynamic reprogramming of cancerous epithelium by the stromal microenvironment enhances the scope of classical cancer pathobiology, and suggests new targets for tumors refractory to current treatments. Moreover, detection of patterns of paracrine responsiveness at early stages might reveal the propensity for progression from premalignant, and preinvasive disease to advanced lesions.

## Results

### Identification of FTExT - a gene expression profile depicting malignant cell response to stromal signaling

**Figure 1:**
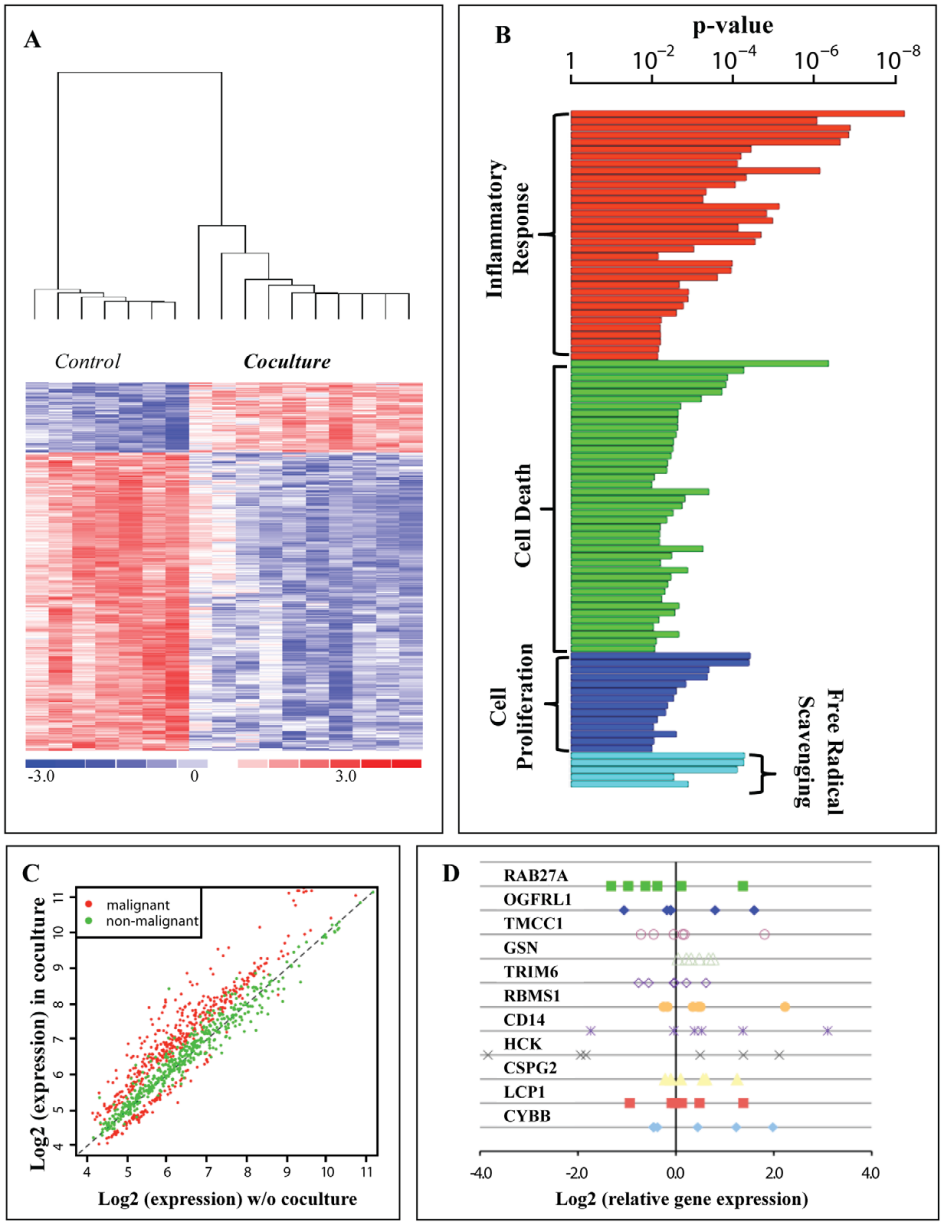
Gene expression changes induced in primary breast cancer cells by fibroblast coculture. **A.** Hierarchical clustering of primary breast cancer cell lines as control monoculture, and in coculture with tumor-derived fibroblasts. Note independent clusters portraying distinctive stromally induced expression patterns in tumor cells reflected by 482 significant probe sets. **B.** Enriched categories of bio-functions represented by 107 probe sets, with >2 fold change in gene expression, designated as FTExT. Bars are grouped and colored according to bio-function categories. Each bar represents an enrichment test p-value of a bio-function. **C.** Analysis of 482 significant probe sets averaged over multiple epithelial cell cultures derived from malignant (red) and nonmalignant breast tissue (green) in the presence (y-axis) or absence (x-axis) of stromal coculture. The black dotted line represents x = y. Tumor cells display greater deviation from this line, i.e. between control and coculture whereas the expression patterns of cocultured nonmalignant epithelial cells shows minimal deviation from control. **D.** Scatter plot of relative transcript levels of 11 FTExT genes measured by QPCR in an independent set of 6 primary breast tumor cell lines cocultured with one of 4 tumor-derived fibroblast (TF) samples. Data represent *ACTB* normalized expression of test gene in cocultured tumor cells relative to control. Note range of expression for each test gene.

A differential expression profile between primary breast tumor cell lines alone (T none) and those propagated in the presence of breast tumor fibroblasts (T+TF) was defined by a significant gene list comprised of 482 probe sets with an adjusted p-value<0.05, where 7 independent primary tumor cell lines were denoted as T, and primary tumor-derived fibroblasts as TF. Expression profiles for 3/7 cell lines were acquired with 2 independent fibroblasts samples. A heatmap, largely reflecting transcriptional repression by tumor-fibroblast coculture (Figure 1A) includes 107 probe sets, with >2 fold change in gene expression (Table S1). This group of genes, which displayed significant negative fold-change in tumor cells, and designated as Fibroblast Triggered Gene Expression in Tumor (FTExT), was investigated further. Upregulated probe sets within the significant gene list did not meet our selection criteria for fold change, and were not considered for further study. Enriched categories of biological functions associated with FTExT genes were determined by Ingenuity Pathway Analysis, and primarily involved inflammatory response, cell proliferation, cell death, and free radical scavenging (Figure 1B, and Figure S1).

Additional approaches to evaluate the diversity of stromal influence, included: (1) comparative analysis of tumor-derived significant gene list (482 probe sets) with array data from stromal-epithelial cocultures isolated from 8 independent nonmalignant breast tissue samples. Gene expression patterns were distinctive between cocultured malignant vs. nonmalignant breast epithelial cells (Figure 1C). Whereas nonmalignant epithelial cells cocultured with corresponding nonmalignant fibroblasts displayed relative uniformity in responsiveness, wide heterogeneity was observed in tumor-derived stromal-epithelial cocultures (2) QPCR analysis of 11 FTExT genes in an independent set of 6 primary tumor-derived epithelial cell lines cocultured with 4 previously untested tumor-derived fibroblasts. Similar to the above-mentioned array data, a gene-by-gene quantitative analysis confirmed induction of changes in test gene expression relative to the housekeeping gene, *ACTB*, within cocultured tumor cells (from 4 matched, and 2 unmatched cocultures) (Figure 1D).

### FTExT variation and association with clinical outcome of primary breast cancer

**Figure 2:**
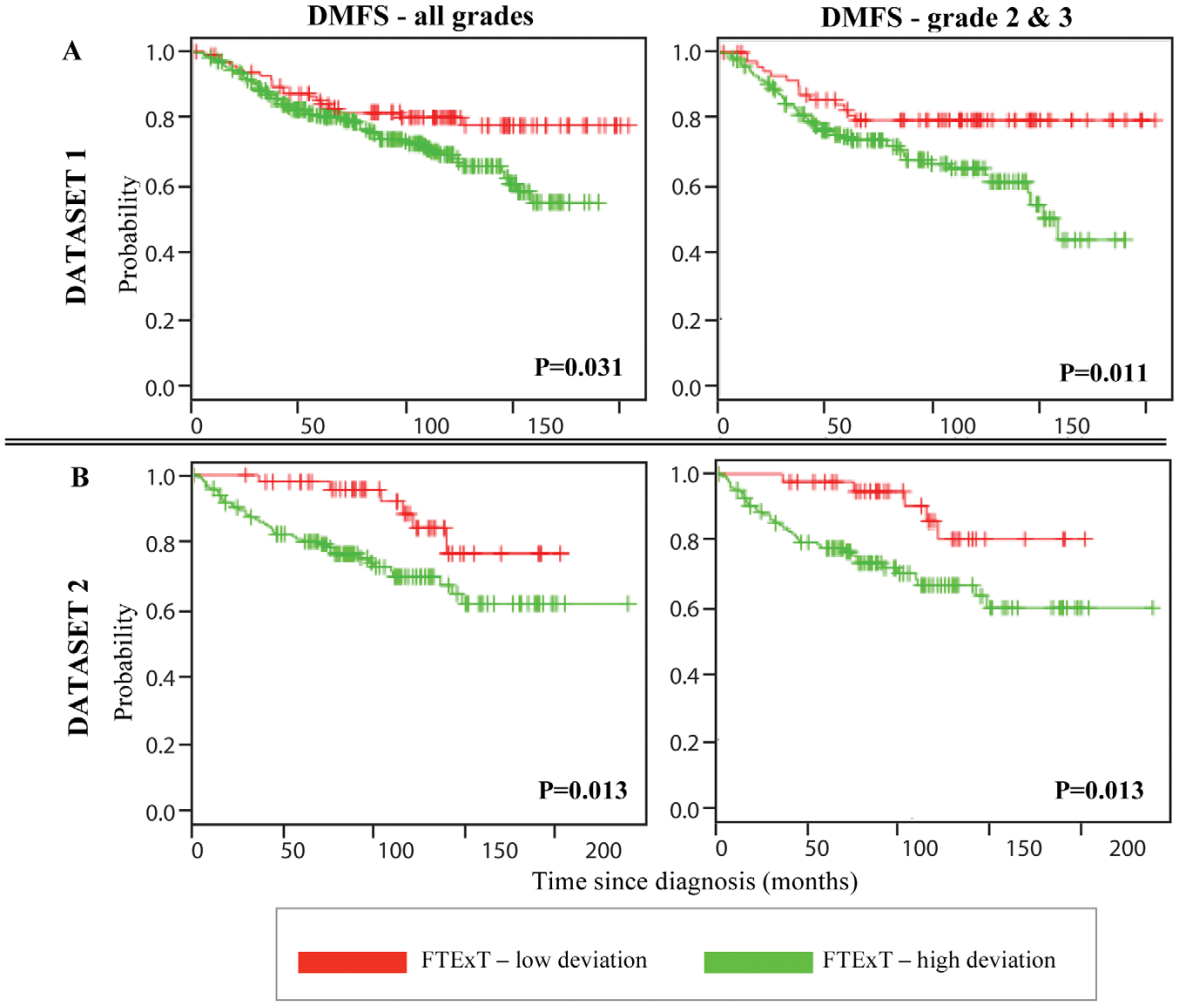
FTExT gene transcript levels and association with clinical outcome. **A.** *Dataset 1 (GSE6532) –* FTExT classifier predicts good vs. poor outcome for the endpoint of distant metastasis-free survival (DMFS) (n = 401, *left panel*). FTExT based stratification of patients diagnosed with grade 2 (intermediate) and 3 (high) tumors (n = 258, *right panel*). **B.** *Dataset 2 (GSE11121)* – FTExT prediction of DMFS in an independent dataset (n = 200, *left panel*). Confirmation of FTExT based stratification of patients diagnosed with grade 2 and 3 tumors (n = 171, *right panel*).

To determine the clinical relevance of phenotypic changes directly induced by stromal crosstalk, the classification power of FTExT (107 probe sets) was tested in conjunction with array data from 2 independent breast cancer datasets. Distant metastasis-free survival (DMFS) was used as an endpoint for patient outcome. In the GSE6532 dataset [22], tumors with poor prognosis displayed significantly greater FTExT gene repression in comparison to those with good prognosis (n = 401; p = 0.031, Figure 2A – left panel). The stratification power of FTExT encompassed tumors of all grades. Moreover, the classifier further improved the subclassification of the subset comprised only of intermediate and high grade tumors (n = 258; p = 0.011, Figure 2A – right panel). In other words, FTExT reliably identified patients who did poorly in situations where traditional histopathology based prognostication was limited in terms of providing an accurate prediction. Reproducibility of the FTExT classifier performance was further demonstrated in an independent dataset [23] – GSE11121 across all grades (n = 200; p = 0.013, Figure 2B - left panel) as well as in a separate analysis of intermediate and high grade breast cancer (n = 171; p = 0.013, Figure 2B – right panel). Notably in all analyses, FTExT was a better independent predictor of the outcome of intermediate and high grade cases compared to conventional prognostic parameters, such as, tumor grade, ER status, lymph node positivity, and tumor size (Table 1).

**Table 1:** Distant metastasis-free survival prediction for intermediate and high grade breast cancer.

Predictor	Log-rank p value	Hazard ratio	(95% CI)
***Dataset 1***			
FTExT	0.01	2.10	(1.17–3.79)
Grade 2 vs. 3	0.86	1.04	(0.62–1.76)
ER	0.62	1.17	(0.61–2.24)
LN	0.07	1.53	(0.95–2.45)
Size (>2 cm)	0.03	1.69	(1.03–2.8)
***Dataset 2***			
FTExT	0.01	3.07	(1.20–7.82)
Grade 2 vs. 3	0.03	1.98	(1.03–3.82)
Size (>2 cm)	0.23	1.44	(0.78–2.65)

Our combined coculture gene expression data and FTExT analysis of primary tumor datasets demonstrated that this stromally induced profile was manifested as a continuum. On one extreme were relatively unchanged patterns of gene expression characteristic of nonmalignant cocultures, shifting to minimal repression of FTExT genes within primary tumors associated with good clinical outcome, while on the other extreme were highly repressed expression profiles within tumors conferring poor patient outcome.

### Induction of a wide spectrum of functional changes in malignant cells by stromal fibroblast crosstalk

In an effort to reveal the biological significance of stromally induced phenotypes as a determinant of clinical outcome, we employed novel primary breast tumor cell lines of varying histologic grade [21] in a variety of functional tests. Reflecting the enriched biofunction analysis of FTExT genes, robust assays were prioritized, where quantifiable endpoints could be employed, and which could be readily conducted in view of limitations in the availability of finite-life stromal cultures. Specifically, changes in the functional endpoints of major cellular programs such as hyperactivated proliferation kinetics; oxidative stress neutralization; and apoptotic cell death evasion were investigated in tumor cells cocultured with fibroblasts derived from malignant or nonmalignant human breast tissue:

#### (1) Reprogrammed cell cycle distribution of cancerous cells

**Figure 3:**
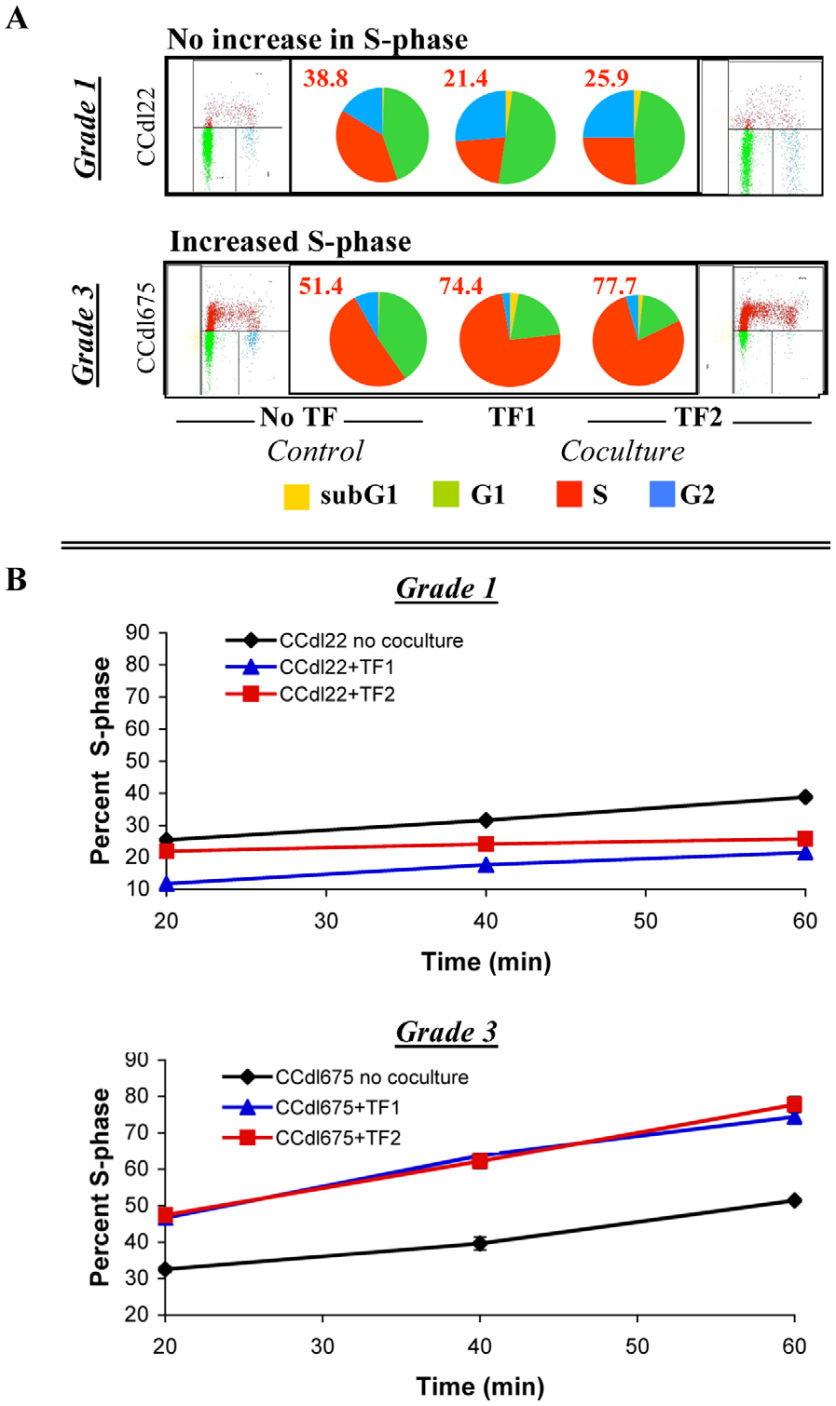
Influence of stromal-epithelial crosstalk on proliferation rate of tumor cells of varying histologic grade. **A.** Cell cycle analysis of primary breast tumor cell lines in coculture with 2 independent fibroblast samples, TF1, and TF2. Pie charts summarize FACS data. Numbers denote percent cells in the S-phase of the cell cycle. Each value is the average of triplicate data points. Representative FACS dot plots are shown for control (left of corresponding pie chart) and TF2-cocultured cells (right of corresponding pie chart). Note varying effects of stromal coculture on tumor cell cycling. **B.** Measurement of S-phase kinetics over time in representative grade 1 and grade 3 cell lines in the presence of TF1 and TF2 cells. Note contrasting effects of stromal cells on the replication rate of cell lines shown. Error bars represent standard deviation of the average of triplicate values. Fibroblast-induced decrease in BrdU labeled cells in grade 1 tumor cells, and a corresponding increase in grade 3 tumor cells was significant at all time points (p<0.005, and p<0.001, respectively).

Ten independent primary breast cancer cell lines under control and TF-coculture conditions were labeled with BrdU, and analyzed by FACS. Cell cycle changes induced by coculture are summarized in Table 2. Dot plots of representative cell lines are illustrated in Figure 3A. The data demonstrated distinctive grade-dependent cell cycle profiles. In response to stromal cell signaling, grade 1 (CCdl22, CCdl68) and grade 2 (CCdl1570) tumor derived cell lines displayed either (a) no significant change in cell cycle distribution compared to non cocultured controls, or (b) induction of cell cycle arrest in the G2/M-phase, indicating activation of a post-replicative/DNA repair checkpoint. In contrast, grade 3 tumor cells (CCdl54, CCdl257, CCdl675), including a high grade ductal carcinoma *in situ* (DCIS)-derived cell line (CCdl1797) and additional grade 2 tumor lines (CCdl66, CCdl329, CCdl1599) displayed a significant increase in the S-phase population, indicative of (a) DNA synthesis/replication defects due to stalling of replication forks, or (b) a faster cell proliferation rate. Notably, the specificity of tumor cell response was common between independent fibroblast samples.

**Table 2:** Cell cycle distribution.

Cell line	No coculture			TF1 coculture			TF2 coculture		
	G1	S	G2/M	G1	S	G2/M	G1	S	G2/M
**No change in %S-phase**									
CCdl22	43.96	38.87	16.53*	49.88	21.49	26.38*	46.91	25.91	24.95*
CCdl68	73.70	20.80	3.75	72.80	23.59	2.9	79.63	16.57	3.63
*CCdl1570*	69.22	24.19	4.5	73.20	20.79	4.49	73.50	17.89	5.90
**%S-phase increase**									
*CCdl66*	57.14	34.91*	4.81	30.03	62.53*	4.52	23.82	69.46*	4.38
*CCdl329*	48.84	36.85*	10.26	32.17	62.37*	4.49	27.75	67.52*	3.51
*CCdl1599*	49.96	36.40*	11.44*	33.35	42.55*	22*	34	43.68*	19.40*
**CCdl54**	45.58	43.75*	10.02	22.34	72.89*	3.69	21.74	73.09*	2.27
**CCdl257**	42.84	45.52*	7.85	28.02	68.84*	2.23	25.03	71.92*	1.07
**CCdl675**	40.08	51.44*	8.06	20.26	74.45*	2.39	16.12	77.79*	4.46
**CCdl1797**	62.94	29.16*	3.01	39.83	52.57*	1.55	39.90	54.54*	1.5

Text style in column 1 indicates pathologist assigned histologic grade of the original primary tumor source of each cell line. Low grade – Regular; Intermediate grade – italics; High grade – bold.

Asterisks represent p<0.005 between control and coculture values within each row.

SubG1 values (range between 0–6%, unchanged by coculture) are included in the % calculation. The combined values of all phases = 100%.

#### (2) Dramatic alterations in the rate of tumor cell proliferation

To confirm that an increased S-phase fraction in response to stromal signaling in grade 3 tumor cells indeed represented a faster proliferation rate compared to grade 1 cell lines, representatives of each group in coculture with 2 independent TF samples were pulsed with BrdU at multiple time points over a 1 hour time period. FACS measurements of the BrdU-incorporating cell fraction over time provided a reliable estimate of the rate of DNA synthesis in tumor cells with and without exposure to fibroblast signaling. Proliferation kinetics of grade 1 (CCdl22) cocultures further confirmed a previously observed fibroblast-induced G2/M arrest. Cell cycle arrest was maintained during the entire experimental time course, leading to a marked decline (up to 70%) in the proportion of BrdU-incorporating cells (Figure 3B). In striking contrast, in grade 3 (CCdl675) cocultures, a dramatic increase of at least 47% and up to 60% occurred in the rate of replication based on the slopes of the growth curves compared to control cells. Induction of a slower or faster proliferation rate in grade 1 and grade 3 tumor cells, respectively, was consistent between independent fibroblast samples.

#### (3) Stroma-assisted protection from apoptotic cell death

**Figure 4:**
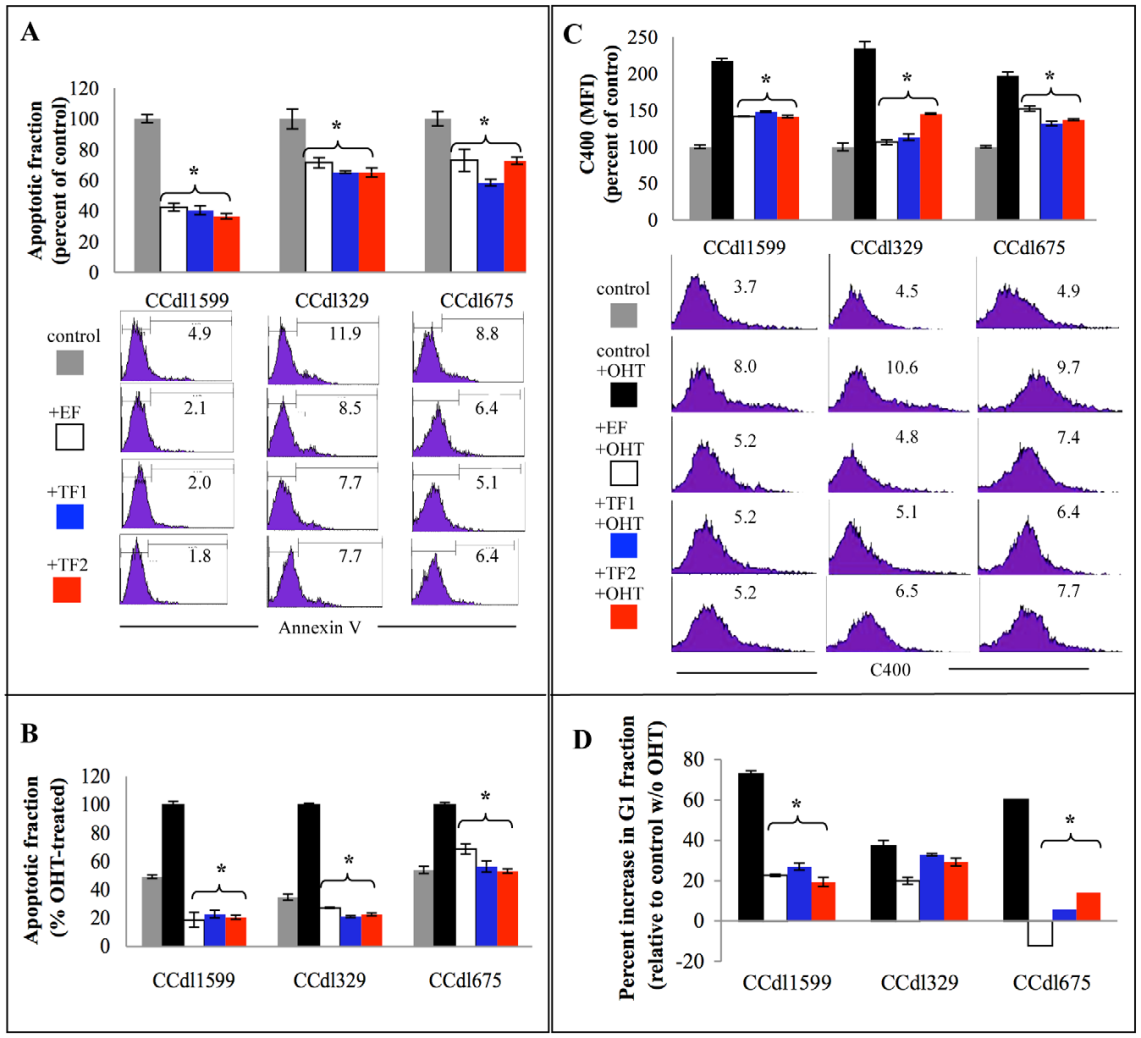
Fibroblast-induced apoptosis evasion, oxidative stress reduction, and inhibition of growth arrest in primary tumor cell lines. **A.** Reduction in baseline apoptotic fraction measured as Annexin-V positive cells by FACS analysis. Three independent tumor cell lines were cocultured with nonmalignant (EF), and tumor-derived (TF1) and (TF2) fibroblasts, and compared to tumor alone control cultures for changes in the apoptotic index. Representative FACS profiles underlying the graphical summaries and percent Annexin-V positive cells are shown. Note significant decrease in the apoptotic fraction of all 3 tumor lines (*p<0.005). **B.** Resistance to OHT-induced apoptotic death in tumor cells cocultured with EF and TF samples compared to drug-treated tumor alone control. Note remarkable repression of OHT-induced cell death by coculture. **C.** Neutralization of cellular ROS levels measured as C400 mean fluorescence intensity (MFI) by FACS analysis. Results are expressed as percent reduction over baseline fluorescence of control tumor cultures vs. cocultures. Representative FACS profiles representing the graphical summaries and MFI values are shown. Note decline in OHT-induced C400 MFI of all tumor lines (*p<0.005). **D.** Coculture induced override of OHT-mediated G1 arrest in tumor cells. Note reduction in percent OHT-induced increase of the G1 population in cocultured tumor cell lines (*p<0.005). Each data point is an average of triplicate values in all panels of the figure. Bars are color coded in panels B and D to represent the following: gray -untreated control, black – OHT-treated control, white – OHT treatment of EF coculture, blue – OHT treatment of TF1 coculture, red – OHT treatment of TF2 coculture.

To evaluate whether stromal signaling enhances survival and propagation of cocultured tumor cells, the ESR1 antagonist, 4-hydroxy tamoxifen (OHT), known to exert its anti tumor effects by oxidative stress induction and subsequent apoptosis, was used to initiate cell death in cell lines, which displayed an increase in percent S-phase in response to coculture. Cell lines derived from both grade 2 (CCdl329 and CCdl1599) and grade 3 (CCdl675) tumors were evaluated. An experimentally derived apoptotic index comparing a single concentration of 10 µM OHT with no OHT control enabled a standardized assay wherein the relative protective effects of stromal coculture could be measured by Annexin-V staining. First, a comparison between baseline apoptosis in tumor cell lines (CCdl675, CCdl329 and CCdl1599) cultured alone or as cocultures with 3 independent fibroblast sources (1 reduction mammoplasty-derived, designated as EF, and 2 tumor derived or TF) demonstrated a stromally induced protective effect (Figure 4A). The apoptotic fraction was reduced by 30–60% in grade 2 cell lines (CCdl1599 - p<0.002; CCdl329 - p<0.001), and by 85% in grade 3 cells (CCdl675 - p<0.005). More significantly, the dramatic spike in OHT-induced cell death was also suppressed by prior exposure of all 3 tumor cell lines to fibroblast coculture. Stromal fibroblasts of both malignant and nonmalignant derivation were equally effective in eliciting apoptosis suppression in tumor cells (p<0.001) (Figure 4B). While it is possible that the variability in the cellular response to OHT evident in coculture is largely a reflection of differential ESR1 activity, our data strongly suggest that growth inducing paracrine signaling in tumor cells could potentially alter sensitivity to hormonal therapy and play a role in the acquisition of a resistant phenotype.

#### (4) Reduction of oxidative stress within tumor cells

The mean fluorescence intensity of C400 staining served well as an indicator of endogenous ROS levels, albeit they are generally low in tumor cell lines compared to inflammatory cell types. A marked decline in OHT-induced ROS accumulation was observed in tumor cell lines (CCdl1599, CCdl329, and CCdl675) cocultured prior to drug treatment (Figure 4C). The average reduction in ROS with coculture was 34% (p<0.0001). All 3 cell lines were consistent in their degree of response to malignant and nonmalignant tissue-derived fibroblasts.

Cell cycle analysis demonstrated an OHT-induced G1 phase arrest in all tumor cell lines tested, which ranged from a 35% to 73% increase in this fraction (p<0.001). Additionally, a mild accumulation in the G2/M fraction was also evident in 2/3 tumor cell lines (p<0.02) in the presence of the therapeutic hormone antagonist. In cocultures of 2/3 tumor cell lines (CCdl1599, CCdl675), the G1-arrested fraction declined significantly despite OHT exposure (p<0.001) (Figure 4D). The protective effect of fibroblast coculture was not as apparent in the CCdl329 line since it displayed relative resistance to OHT treatment as indicated by the lowest increase in the G1 fraction – suggesting that the G1/S checkpoint may be defective in these cells. Interestingly, despite the induction of only a mild G1 arrest, CCdl329 cells were responsive to OHT-induced apoptosis (Figure 4B). Such differential responses to OHT suggest other sources of variability in breast tumors, which further modify the outcome of tumor-stroma interactions, and reflect additional sources of heterogeneity.

Taken together, our data demonstrates that fibroblast coculture facilitated a parallel reduction in 3 independent but closely related parameters of OHT-induced damage: ROS accumulation, G1 arrest, and apoptotic cell death within an aggressive subset of primary breast tumor derived cell lines. However, despite tumor promoting responsiveness displayed by these cell lines, marked variation was evident. CCdl329 cells effectively evaded OHT-induced apoptosis even though G1 arrest in the presence of the drug was only partially overcome by coculture. In contrast, CCdl675 cells were relatively deficient in surmounting the apoptotic burden despite the observed ROS reduction and a striking decline in the G1 and G2/M arrested fraction, consistent with our observations of deficient repair culminating in increased genomic instability (data to be published elsewhere).

### Stromal fibroblast induced functional impact is reversible

**Figure 5:**
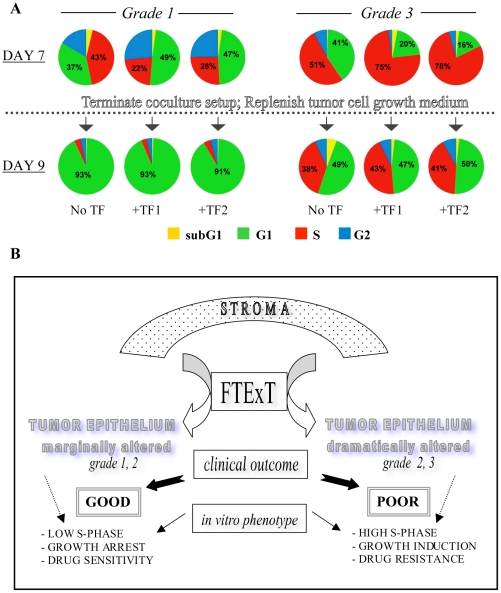
Reversibility of coculture-induced changes in cell cycle. **A.** Comparative cell cycle profiles of grade 1 and grade 3 tumor cells after 7-day fibroblast coculture, followed by a 48 hr period without coculture. Note marked reduction in the G2 population of the grade 1 cell line upon termination of coculture conditions. Both control and test populations display signs of G0/G1 arrest due to culture confluence. In the grade 3 tumor line, post coculture cell cycling returns to control profiles. Numbered pie chart segments indicate the percentage of cells in G1 and S phase. SubG1: yellow; G1: green; S: red; G2/M: blue. **B.** ***The FTExT model*** *:* through the application of live interactive cocultures of stromal fibroblasts and primary tumor-derived epithelium of varying histologic grade, FTExT offers novel insights into the biological basis of grade-associated stratification of primary breast cancer. It proposes that cell lines derived from well-differentiated tumors associated with good clinical prognosis recapitulate their functional characteristics in the presence of stromal fibroblasts *in vitro*. Similarly, tumor cells derived from poorly differentiated malignant lesions retain the contextual ability to respond to the same paracrine influence in a manner corresponding to cancer aggressiveness and unfavorable patient outcome. Such authentic model systems are an essential tool for advancing the goals of research in cancer biology.

Finally, to evaluate the reversibility of distinctive and deterministic endpoints of stromally induced growth aggressiveness, tumor cells were analyzed over a short-term recovery period for reversal of cell cycle changes, and induction of cell death, after coculture conditions were discontinued (Figure 5A). Removal of the stromal influence from grade 3 (CCdl675) cocultures reversed the striking S-phase increase over control during the 48 hr test period. However, enhancement in apoptotic cell death was not detected during this period (data not shown), confirming that coculture induced increase in the BrdU-labeled fraction of tumor cells represented a larger cycling population, instead of accumulation in S-phase due to replication-arrest and fork stalling. In the event of the latter possibility, termination of coculture would have led to cell cycle progression in tumor cells harboring an unreplicated set of chromosomes, culminating in mitotic catastrophe and cell death. In grade 1 tumor cells (CCdl22), which responded to coculture by displaying an average increase of 64% in the G2/M arrested population relative to control (p<0.001), cell cycle re-entry was observed upon fibroblast removal. However, as the culture reached confluence within the experimental time frame, a subsequent G0/G1 arrest was apparent in both control and cocultured grade 1 cells, strongly suggesting that (a) these tumor cells were likely DNA repair proficient, as removal of stromal effectors surmounted the G2 checkpoint, and that (b) the growth potential was intact, but responsive to negative regulation by paracrine signaling. Had the G2/M checkpoint not been reversible but due instead to the onset of mitotic arrest in cocultures, it would have resulted in apoptotic cell death, detectable as a substantial increase in the subG1 population within the test period.

Overall, the experimental findings in this study are consistent and supportive of differential clinical outcome data derived through correlative FTExT profiling analysis of primary breast cancer. Together, they provide a novel conceptual framework for the significant association between the FTExT expression pattern in moderately and poorly differentiated or grade 2 and 3 tumor respectively, and the role of the stroma in the induction of rapid growth and poor clinical outcome. In contrast, checkpoint induction in well differentiated or grade 1 tumor cells mediated by stromal signaling corresponds to the observed gene expression pattern of primary tumors, which confer a good clinical outcome (Figure 5B).

## Discussion

Towards a complete picture of cancer development and progression, a comprehensive delineation of underlying sources contributing to the manifestation of malignant phenotypes and their heterogeneity, is critical. In previous studies we have uniquely identified intrinsic phenotypes reflecting histologic tumor variability but independent of contextual influences, such as stromal induction [21]. By employing experimental coculture in conjunction with novel primary tumor cell lines, here our approach has identified FTExT, a programmed response to stromally secreted factors. Instead of a stromal impact that uniformly promotes or inhibits all tumors, our data demonstrate the significance of variable epithelial responsiveness in disease outcome. Together with correlative analyses of clinical tissue, such focused yet broad-scope investigative strategies to define the functional framework of cancer phenotypes could substantially improve the range and accuracy of druggable target identification.

The FTExT profile generated through experimental coculture portrays stromal regulation of major biological programs, suggesting a key role for stroma-assisted promotion of early hallmarks of cancer encompassing evasion of regulatory circuits, such as immune surveillance, oxidative stress, and metabolic deficiency [24], in addition to the hallmarks of advanced cancer [25]. Consequently, the FTExT expression profile was found to be associated with a poor clinical outcome in two independent datasets, representing over 600 cases. Consistent with this correlative clinical data, we have further demonstrated, that the downstream functional effects of stromally induced transcriptional changes were dramatically variable between primary breast tumor cells of divergent histopathologic derivation. In aggressive breast cancer cells, tumor-promoting effects were evident as an increased ability for oxidative stress neutralization, apoptosis evasion, and rapid cell cycling. In contrast, non-aggressive cells displayed the consequences of negative growth regulation or tumor inhibitory effects in response to paracrine interaction. It can thus be concluded that FTExT is a functionally validated surrogate for measuring the impact of stromal signaling on malignant epithelium within cancerous breast tissue. Based on a consistent pattern of responsiveness irrespective of the source of breast fibroblasts, our data suggest that contrasting responses of malignant cells are characteristically ‘hard wired’ into the tumor cell genome. We have previously demonstrated that 2/3 novel high grade cell lines in this study cluster with other well-known luminal cell lines [21]. In contrast, our low and intermediate grade cell lines cluster apart from all other established cell lines, suggesting the possibility that the unique responsiveness of such novel breast cancer cell lines to stromal signaling reflect a distinct molecular makeup. Reflecting a model of ‘effective cooperativity’, neighboring stroma within malignant tissue plays the role of a key accomplice, but only to the tumor epithelium armed with significant biological derangement. Consequently, treatment strategies could be better informed by the early detection of those responsiveness patterns wherein stromal interaction promotes tumor survival and growth at the expense of the host.

In terms of relevance to histologic grade, this is the first report of a functionally mediated, stromally induced molecular component associated with global prognostic profiles of grade 2 and 3 tumors. Grade is an important histologic determinant of patient management. More recently, in attempts to eliminate the subjectivity underlying its histologic assignment, a molecular grade gene index has been implemented [26], [27] based on genes, which are predominantly associated with tumor cell proliferation [28]. The distinctive components of the FTExT signature, and their role in distinguishing those grade 2 and 3 tumors that do not confer an expected poor outcome, could further assist in improving the reliability of tumor grading and prognostic assessment. Our model maintains that in indolent breast tumors, where differentiation signaling is relatively intact, stromal fibroblasts and malignant cells are not engaged in growth promoting ‘cooperation’. On the other hand, the loss of responsiveness to differentiation cues in grade 3 and some grade 2 lesions induces a dramatic shift in the epithelial-stromal dialog towards tumor promotion. It could be argued that generally at advanced stages, grade 3 cancer displays low stromal cellularity, and therefore crosstalk between malignant cells and other components of the tumor, is marginal. In such tumor cells, repression of FTExT genes might be an inherent phenotype independent of stromal signaling. While this possibility cannot be categorically eliminated at this time, our experimental data demonstrating that cells derived from grade 3 tumors harboring phenotypes of poor prognosis [21] are indeed promoted further in their aggressive attributes through paracrine interaction, are strongly supportive of a stromal role. In an alternate scenario, the molecular profile characteristic of high histologic grade is established early in tumorigenesis partially through stromal interaction, and maintained thereafter. An example closely supporting our model of interactive cooperativity is that of dermal fibroblasts, which repressed the growth of non-metastatic melanoma lesions while proliferation of metastatically competent melanoma cells was stimulated in their presence [29]. In this light, it could be speculated that FTExT might play a role in the increased risks associated with mammographic breast density [30], [31] based on the rationale that overabundance of ‘cooperative’ fibroblasts associated with responsive premalignant or malignant cells leads to more aggressive lesions. This is also consistent with the finding that carcinomas that exhibit a greater stromal reaction manifested microscopically as a “desmoplastic response” are associated with higher histologic grade and poor patient outcome [32].

Intriguingly, considerable variability in signal induction and/or elicited response appears to occur between cancer-associated fibroblasts from different tumor types emphasizing the importance of specificity in the crosstalk between stromal and malignant cells of diverse tissue origin. With regard to 26 probe sets common between the coculture transcriptional profiles in our study and myeloma cells cocultured with bone marrow derived stromal cells [33], 25 probe sets displayed suppression in the FTExT profile, but in remarkable contrast their expression was induced in myeloma cocultures. Such inter-study comparisons are invaluable and underscore the importance of well-defined model systems for the derivation of clinically relevant data for each tumor type. As a striking example of tissue specific effects, although loss of function for PTEN, a FTExT gene, is a well-known indicator of tumor aggressiveness [34], this carcinoma-associated tumor suppressor gene appears to be overexpressed in myeloma patients with poor clinical outcome [33]. Similar contrasting patterns of expression were noted underlying the role of FTExT genes: CYBA, GNAI2, GRN, JAK3, TRADD, BCL2A1, BPGM, and CD48 in carcinomas of the breast and other tissues, in comparison to myeloma. However, both in myeloma as well as in breast cancer, signatures portraying altered functionality due to paracrine exchange between stromal and tumor cells, were characteristic of poor clinical outcome, suggesting similar underlying pathways albeit transcriptionally distinctive. The fact that although stroma of different tissues regulate a closely similar set of genes, but in opposing directions in two tumor types, might reflect contrasting functions of their precursor cell types - epithelium vs. B lymphocytes, in this case. This is a critical finding in and of itself, emphasizing careful consideration prior to data extrapolation from one cellular subset or tissue type to another. Thus caution must be exercised in employing irrelevant cell types and their modulating microenvironment in highly cost-intensive drug discovery efforts. In a similar vein, it is notable that the FTExT signature includes several genes characteristic of immune response. While the induction of these genes in tumor tissue confers a favorable prognosis in breast cancer patients [35], [36], it is consistent that their repression would have the reverse effect as demonstrated by our data and by others [37].

Our approach complements profiling strategies for identifying the biological basis of the clinical heterogeneity of human malignancy in general, and breast cancer in particular, but differs in the striking innovation of engaging functional cell models spanning the clinicopathologic spectrum of this disease. The need for hypothesis validation through gene-by-gene manipulation of cellular and rodent models, severely limited in the representation of the full range of human cancer pathology, is thus circumvented. Portrayed as dynamic paracrine interactions, which lead to the clinical, molecular, and functional heterogeneity of breast cancer, our data present novel opportunities for tumor targeting. Thus, therapies that enable the disruption of cancer-promoting interactions in the malignant microenvironment could serve as an important adjunct to current approaches, which only target malignant subpopulations to a variable degree. The observation that secreted stromal factors are remarkably effective in modulating tumor phenotypes brings a long desired goal in the field closer to fruition, whereby the detection of tumor associated markers in body fluids could be facilitated in the future. Concerted efforts towards the identification of stromal signals that fuel dramatic changes represented by the FTExT profiles of tumor cells should be a priority for impeding such crosstalk in high risk tumors, such as triple-negative breast cancer. In this regard, previous investigations of microdissected fibroblasts and tumor cells within archived breast tumor tissue have indeed demonstrated characteristic features of stromal and epithelial cell lineages [38], but not distinguished between inherent and induced characteristics of each subset, thus only partially addressing their respective role in the overall tumor phenotype. On the other hand, an inducible model of squamous cell carcinoma highlighted the importance of tumor-stroma interaction and identified the temporal induction of ß1 integrin as a potential nexus involved in Ras-driven tumor progression [39], but functional dissection of inter-tumor heterogeneity was not a major goal. Among those that could benefit considerably from targets manifested early in tumorigenesis are women that harbor DCIS, currently the most common diagnosis in the Western world associated with breast related symptoms [40]. Since DCIS is a preinvasive lesion, and often indolent, it presents the clinician with the dilemma of distinguishing between those that will recur with invasive disease, thus requiring immediate aggressive therapeutic measures, from those that will not. As suggested by the FTExT signature, stromally induced cues might promote the release of *in situ* tumor cells from the confines of the basement membrane, as well as support subsequent steps in tumor survival and expansion. Thus, detection of FTExT-related aberrations within diagnostic biopsies could improve clinical decision-making, and offer clear opportunities for personalized patient management.

## Methods

### Cell culture

The development of spontaneously immortalized high grade, and non-spontaneously immortalized, hTERT-transduced low and intermediate grade primary breast cancer cell lines has been previously described [41]. All primary tumor cell lines were maintained in MCDB 170 growth medium supplemented with 2% FBS. Collection of surgical discard tissue and of non-malignant breast epithelial cells as random periareolar fine needle aspirates (RPFNA) from unafflicted contralateral breast tissue of patients undergoing surgical procedures for benign or malignant disease was reviewed and approved by the California Pacific Medical Center Institutional Review Board. All human subjects signed informed consent forms prior to sample collection. Microarray analysis conducted on samples without patient identifiers was approved by the Stanford University Human Subjects Research Compliance Board. Epithelial cells within RPFNA cells were propagated as previously described [42]. Fibroblast cultures derived from mechanically and enzymatically dissociated surgical discard primary breast tumor, or reduction mammoplasty tissue, were routinely propagated in DME+10% FBS. In the coculture set up, epithelial cells were seeded in 6-well plates, and second to third passage fibroblast cells in 0.4 µm inserts with hanging geometry (BD Biosciences, Franklin Lakes, NJ) at a 3∶1 ratio in a common pool of MCDB 170 growth medium for 3-day harvests. Controls were comprised of each epithelial sample maintained in the absence of fibroblast-seeded inserts under the same culture conditions.

### Array analysis and data preprocessing

Gene expression analyses were performed on epithelial tumor cells removed from 3-day coculture with stromal fibroblast inserts within 6-well plates, and compared to non-cocultured epithelial tumor cells in independent 6-well plates processed simultaneously. Tumor cell RNA was isolated with the RNeasy kit (Qiagen, Valencia, CA) and applied towards global gene expression profiling using Human Genome U133 Plus 2.0 arrays (Affymetrix, Santa Clara, CA). Raw CEL files were processed using Affymetrix Expression Console by the robust multichip analysis (RMA) method with default parameters [43]. Significant genes were inferred using linear regression models in the limma R package of Bioconductor [44]. Expression of each gene was fitted into a linear model of base-line expression, an individual tumor cell line effect and a coculture effect. Genes with significant coculture effects were selected as signatures between tumor only and cocultured samples. Statistical significance was adjusted by multiple hypotheses testing with the Benjamini-Hochberg procedure [45]. Fold changes between tumor cells with and without coculture were calculated from the gene expression data adjusted by removing individual tumor cell line effects. The microarray data in this report is MIAME compliant and the raw data can be accessed under GSE27018 in the NCBI Gene Expression Omnibus database. Biological functions of the significant genes were derived by Ingenuity Pathway Analysis tools (www.ingenuity.com).

### Classification and survival analysis

A classifier was built upon 107 significant probe sets with fold changes >2 for two classes, like-T (similar to tumor cells only) and like-T+TF (similar to tumor cells cocultured with fibroblasts). The classification power was tested over two independent data sets, GSE6532 (n = 401) and GSE11121 (n = 200). A classification score of each sample was calculated as the projection of an expression profile to the first principle component of the standardized expression matrix of a training set, the FTExT data. The class of a tested sample was determined as the closest class in terms of Gaussian distance of the sample's classification score to the centroid of each class in the training set. Univariate analyses of the predicted class, and conventional prognostic factors were performed with the Cox proportional hazard regression model. Survival of patient groups was visualized with Kaplan-Meier curves, and compared using the log-rank test.

### Real time quantitative PCR (QPCR) analysis

RNA from 6 independent sets of tumor epithelial-stromal cocultures was used to validate expression patterns of 11 top SAM genes. Total RNA was isolated using the RNeasy kit (Qiagen) from tumor cells cultured alone or as cocultures with tumor fibroblasts for 7 days. RNA concentrations were determined by NanoDrop 2000 (Thermo Fisher Scientific, Waltham, MA). cDNA was synthesized and analyzed as before [21] by an Applied Biosystems 5700 Sequence Detection System (Foster City, CA). The Ct values of test genes were normalized to the expression of the housekeeping gene, *ACTB*, within each sample to represent log base 2 fold increase or decrease in test gene expression over no coculture controls. Primer sequences for test genes are listed in Table S2.

### Quantitation of endogenous reactive oxygen species (ROS)

Endogenous cellular ROS accrual was determined as previously described [46] using live tumor cultures loaded with the fluorogenic dye, Carboxy-H2DCFDA (5-(and-6)-carboxy-2′7′-dichlorodihydrofluorescein diacetate), also known as C400 (Invitrogen, Carlsbad, CA) for 1 hr. After dye removal, cells were counterstained with propidium iodide (PI), and intracellular oxidation of C400 was measured by FACScan (BD Biosciences) with the FL1 filter. Experiments were done in triplicate and 10,000 cells were acquired for each sample. ROS activity was expressed as mean fluorescence intensity (MFI) of the C400 dye. MFI of PI-negative (non-necrotic) cells was corrected for autofluorescence.

### Analysis of apoptotic cell death

Apoptosis was induced in tumor cells after removal from coculture wells by a 24 hr treatment with 10 µM 4-hydroxytamoxifen (OHT). Parallel controls were maintained for 24 hours without OHT. For quantitation of OHT-induced apoptotic cell death, cultures were harvested and stained with Annexin V-FITC and PI (BD Biosciences) according to the manufacturer's protocol. Cells diluted in binding buffer were analyzed by FACScan and quantified by CellQuest software (BD Biosciences). Annexin-V positive cells were measured as early (PI-negative) and late (PI-positive) apoptotic cell fractions. Each measurement was performed in multiple replicates. Results were expressed as percent of control (OHT-treated without prior coculture).

### Cell cycle kinetics

Tumor cells were exposed to malignant or nonmalignant breast tissue-derived fibroblasts, or maintained as control cells without coculture. At the end of the coculture period, fibroblast-bearing inserts were removed and tumor cells were pulse-labeled with 10 µM BrdU for varying time periods (20–60 minutes), stained with anti BrdU (Santa Cruz Biotechnology, Santa Cruz, CA), FITC-conjugated secondary antibody (Invitrogen), counterstained with PI, and analyzed by FACScan.

## Supporting Information

Figure S1 Top 25 enriched bio-function categories for significant probe sets suppressed in primary breast tumor cells cocultured with stromal breast fibroblasts. Bar represents a p-value of the most significant function of a category.
(DOC)

Table S1 Identity, and fold-change in FTExT gene expression
(XLS)

Table S2 Primer sequences for genes tested by QPCR
(DOC)
