# Supplementary figures and images for: Distinctive Responsiveness to Stromal Signaling Accompanies Histologic Grade Programming of Cancer Cells
Source: PLoS One. 2011 May 19;6(5):e20016. doi: 10.1371/journal.pone.0020016 (PMC3098270; doi:10.1371/journal.pone.0020016)

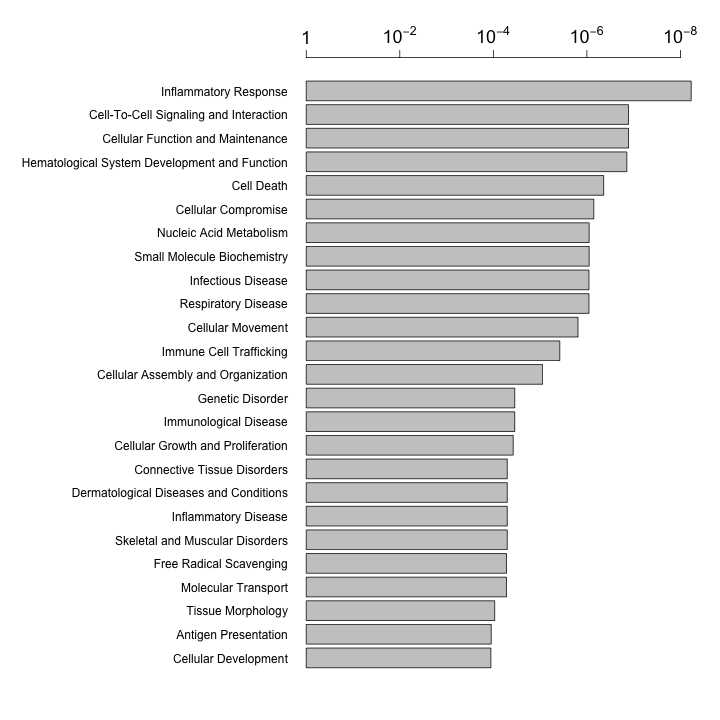
 **FIGURE S1**

Supplement: Figure S1 — Top 25 enriched bio-function categories for significant probe sets suppressed in primary breast tumor cells cocultured with stromal breast fibroblasts. Bar represents a p-value of the most significant function of a category. (DOC) [file pone.0020016.s001.doc]
