# Supplementary material for: Distinctive Responsiveness to Stromal Signaling Accompanies Histologic Grade Programming of Cancer Cells
Source: PLoS One. 2011 May 19;6(5):e20016. doi: 10.1371/journal.pone.0020016 (PMC3098270; doi:10.1371/journal.pone.0020016)
Supplement: Table S2 — Primer sequences for genes tested by QPCR (DOC) [file pone.0020016.s003.doc]

**TABLE S2**

**Oligonucleotide sequences for QPCR primers**

RAB27A forward: GAAAATACCCGCCAACGAC; RAB27A reverse: ACAGGGTAGAGAACCGCTTGT

OGFRL1 forward: GCGAGGGTGCCTGTCCG; OGFRL1 reverse: CTGACCCCGCCGAGCAG

TMCC1 forward: GCAAAGGTGACTGGCTTCAG; TMCC1 reverse: AGAGTCCTTCAGGTTGGGGA

GSN forward: GAACACCCCGAGTTCCTCAA; GSN reverse: GCAGATTTCCGTTCCTCAGC

TRIM6 forward: TGTGGGTCCATCCGTTCAAC; TRIM6 reverse: TCAGAGCAATCCAAGGCACC

RBMS1forward: GAAAGGGAGAGGCAGGAGAG; RBMS1 reverse: CTGGGACCAGAGACTGCTTG

CD14 forward: GGACACTGCCAGGAGACACA; CD14 reverse: TCACCTCCCCACCTCTCTTC

HCK forward: AAAGTCAGTTTCCCGGCACTG; HCK reverse: ATGTATTGCCTCCGACCTGGA

CSPG2 forward: TTCTTCTCGCTGAGTCTCCTCCT; CSPG2 reverse: GCTCCTGTCCACAACACCTAATG

LCP1 forward: TAACCCCTTTCACCATTCAGGAA; LCP1 reverse: CAGCAAACAACCCAATCTTGATG

CYBB forward: AACTTCTTGGGTCAGCACTGGCA; CYBB reverse; GAACTCTTGAGCAGCACGCA
